# Towards an optimal hybrid solar method for lime-drying behavior

**DOI:** 10.1016/j.heliyon.2020.e05356

**Published:** 2020-10-27

**Authors:** Suherman Suherman, Hadiyanto Hadiyanto, Evan Eduard Susanto, Shesar Anis Rahmatullah, Aditya Rofi Pratama

**Affiliations:** Department of Chemical Engineering, Faculty of Engineering, Diponegoro University, Tembalang, Semarang, 50275, Central Java, Indonesia

**Keywords:** Lime, Drying, Hybrid solar dryer, Dryer efficiency, Thin-layer modeling, Agricultural engineering, Food engineering, Food technology, Postharvest food processing, Thermal food processing

## Abstract

Lime is one of the most commonly consumed medicinal plants in Indonesia, which must be dried to preserve its quality, but mostly by using traditional, ineffective drying method. Therefore, this study aims to investigate lime drying process a hybrid solar drying method. The hybrid solar dryer consisted of a solar dryer and Liquefied Petroleum Gas as the supplementary heater. The drying process was conducted until there was no significant weight decrease, with the drying temperature of 40, 50, 60, 70, and 80 °C. Thin-layer modeling and quality analysis were also conducted. The experimental results indicated that 5 h was required to sufficiently dry the lime at 80 °C, while drying at 40 °C took 24 h to finish. The drying rate curve of lime suggested that lime drying mostly happened during the falling-rate period. Moreover, the average efficiency of the hybrid solar dryer ranged from 5.36% to 38.61%, which increased with temperature. From the 10 thin-layer drying models used, the Wang and Singh model was the most suitable to describe the drying behavior of lime. The effective diffusivity values of the limes and the activation energy value during hybrid solar drying were within their respective acceptable range for agricultural products. However, as the drying temperature was increased from 40 to 80 °C, the total phenolic content and vitamin C content decreased, from 87.3 to 27.8 mg GAE/100 g dry limes and 0.118 to 0.015 ppm, respectively. It can be concluded that hybrid solar dryer is able to sufficiently dry the lime, with acceptable drying time and dryer efficiency, although using high drying temperature will decrease the quality of dried lime. Further modifications and improvements to the hybrid solar dryer are required to maximize the quality of dried lime while still maintaining fast and effective drying process.

## 1 Introduction

Indonesia is a tropical country with a lot of medicinal herbs that have been utilized to treat disease. One of which is lime, which has been widely used as traditional medicine by Indonesians [1]. Lime (*Citrus aurantifolia* sp.) has a smooth fruit surface, greenish-yellow in color and has a thin skin. Several major flavor components of lime include limonene, citral, geranial, 1,4- cineole, α-terpineol, and many more [2]. Researches have shown that lime has many beneficial functional components, such as phenolic, limonoids, flavonoids, and polysaccharides. In some regions in East-Asia and China, lime peels have been used for traditional herbal medicine as antioxidant, antibacterial, anti-inflammatory, and anti-tumor [3].

In 2018, Indonesia produced 2.4 tons of both orange and lime, which saw increase from 2017 by 0.24 tons [4] Although Indonesia produces large quantities of lime, applications are currently limited because of the damage caused during the distribution process. Lime is mostly used while it is fresh, and fresh lime has high moisture content. This means that it is vulnerable to spoilage/damage due to infestation by insects, bacteria, or fungi. Therefore, in order to preserve lime, it must be dehydrated, drying being one of the most commonly used method [2, 5, 6]. Some small–medium enterprises in Indonesia have implemented lime drying; however, they make use of the traditional sun drying method, resulting in a long drying time—approximately 1 week. In addition, the quality of dry lime is low because of exposure to unfavorable environmental conditions, such as dust and wind. Moreover, during the rainy season, lime enterprises stop production because lime cannot be dried during the said time, which negatively affects the lime farmers [7].

Although drying holds an important role in lime production, relatively few studies have discussed lime drying. A study examining ohmic and conventional drying for oranges has found that the wet-basis moisture content of orange decreases from 62% to 55% when dried for 30 min at a temperature of 70 °C and that ohmic drying helps with vitamin C retention [8]. Another study examining the kinetics of orange peels with a forced-convection solar dryer suggests that the dryer in question reduces orange-peel wet-basis moisture content from 80% to 28% in 4 h [9]. Other research examining the effects of sun drying, hot-air drying, and freeze drying on the phytochemical compounds and antioxidants of citrus fruits suggests that freeze drying can preserve more phytochemical compounds compared with hot air and sun drying [10].

It has been widely known that solar drying is one of the simplest and cheapest drying methods, especially in developing countries. Similar to traditional drying, it uses solar radiation; however, the products in question are placed in a confined chamber with transparent walls that intensify the light. This method is eco-friendly, and the dried products are protected from contamination; moreover, the dryer designs are flexible based on the need and available sources and the procedure is relatively simple to perform and cost-effective. However, the solar drying method still relies on the weather, which reduces its effectiveness [11, 12]. To solve this problem, as well as to speed up the drying process, an auxiliary heater can be integrated into the solar dryer, which increases efficiency and eliminates the weakness of the pure solar dryer, namely, relying on the sun. These kind of dryers are called “hybrid solar dryers” due to additional means of heating alongside solar energy. The auxiliary heaters may vary, from biomass, Liquefied Petroleum Gas (LPG), thermal water tank storage, and many more [13].

Several studies have discussed the implementation of hybrid solar drying for food drying, using LPG as the auxiliary heater. Anum et al. [14] studied the drying behavior and performance evaluation of gas fired hybrid solar dryer. They found that the dryer is able to reduce the moisture content of cabbage, onion, and ginger to their respective standard limit with the drying time of 13 h, respectively. The overall drying efficiency of the hybrid solar dryer is 42.79%. Meanwhile, Murali et al. [15] designed and evaluated the solar – LPG hybrid dryer for drying of shrimps. They found that in 6 h, the dryer managed to reduce the shrimp's moisture content from 76.71% to 15.38% wet basis, with initial shrimp weight of 50 kg. The drying efficiency varied between 24.21 to 37.09%, with the average value of 29.93%.

To the best of the authors' knowledge, lime drying using a hybrid solar dryer has yet to be examined. Another problem is that, despite the fact that hybrid solar dryer has been widely used for many agricultural products, no studies about lime drying can be found. Therefore, this paper aims to investigate the performance of a hybrid solar dryer for limes. The hybrid solar dryer in question consists of an indirect-type solar dryer complemented with LPG as an additional heat source. Several factors are examined, such as moisture content curve, drying rate, and dryer efficiency, to assess the performance of the dryer. Moreover, thin-layer modeling is conducted to determine the best drying model for describing the behavior of lime drying. Finally, quality analysis is carried out, specifically total phenolic content and vitamin C content analysis, to identify changes in lime quality during the drying process.

## 2 Materials and methods

### 2.1 Materials and tools

The main component required for this experiment was lime, which was obtained from the local market. Other necessary materials included the following: Folin–Ciocalteau reagent, ascorbic acid, gallic acid (from Sigma-Aldrich), aquadest, and sodium carbonate. These materials were used for the phenolic content analysis. In addition, the measurement tools used in this experiment were Krisbow Electronic Kitchen Scale 5 kg Slim Plate digital scale (made in Indonesia), Krisbow KW0600561 Digital Temperature and Relative Humidity meter (made in Indonesia), BONAD SM206 Solar Power Meter (Made in China), Krisbow KW0600562 Flexible Thermo Anemometer Unit (Made in Indonesia), Digital Multimeter Fluke 117 True RMS Original (Made in Indonesia), Memmert Universal Oven UN55 (made in Germany), and OPTIMA SP-300 Spectrophotometer (made in Indonesia). The details and specifications of the measurement tools are shown in Table 1.

**Table 1:** Details and specifications of measurement tools [16].

No	Measurement tools	Measured parameter	Accuracy
1	Digital scale	Lime weight	±0.1 g
2	Solar power meter	Solar intensity	±0.1 W/m^2^
3	Temperature and relative humidity meter	Ambient temperature Ambient relative humidity	±0.01 °C ±0.01 %
4	Anemometer	Drying air velocity	±0.01 m/s
5	Multimeter	Blower's voltage Blower's electric current	±0.1 V ±0.1 A
6	Oven	Initial moisture content of lime	-
7	Spectrophotometer	Absorbance of lime solution	±2 nm

### 2.2 Description of hybrid solar dryer

**Figure 1:**
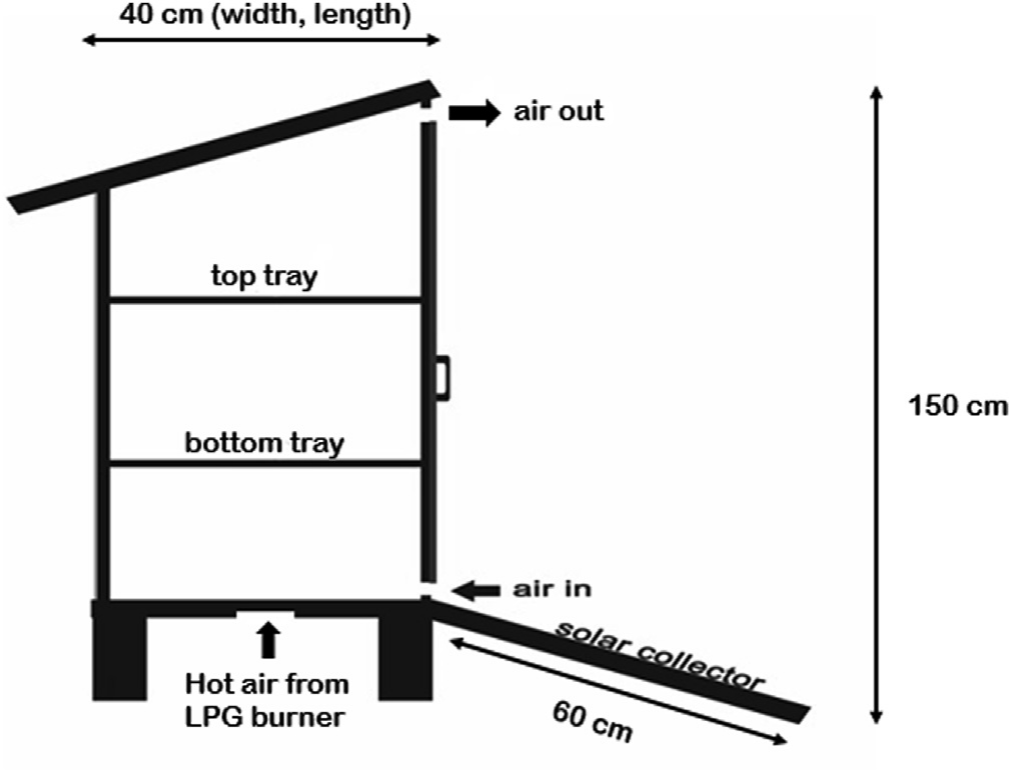
Schematic drawing of drying chamber, side view.

**Figure 2:**
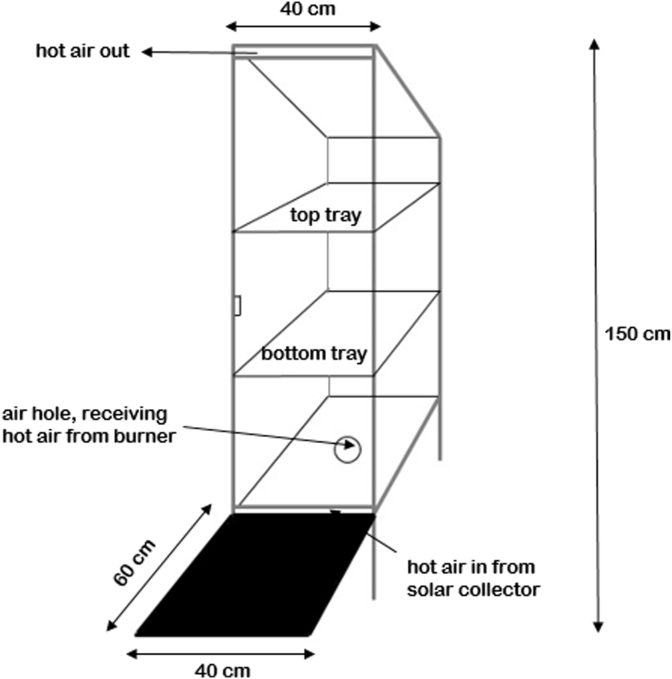
Schematic drawing of drying chamber, front view.

Figures 1 and 2 show the schematic drawing of the drying chamber from the side and the front, respectively. The drying chamber is 40 cm long, 40 cm wide, and 150 cm tall. The outer frame of the drying chamber was made from aluminum, a good heat-conducting material while also being lightweight and relatively easy to obtain. The drying-chamber walls were made from glass. At the bottom front of the drying chamber, a solar collector with 60 cm long and 40 cm wide was jointly connected with the bottom part of the drying chamber. The solar collector was inclined roughly 22.5°, facing upwards. The surface of the solar collector was made from black thermoplastic rubber, which also acted as solar radiation absorber [15]. Two drying trays can be found inside the chamber, and the front wall can be opened and closed for the easy removal of the limes. The drying-chamber roof was tilted downward at the back, at 30°, facing upwards. This was to prevent water vapor from re-entering the upper air duct of the drying chamber. There were two hot air inputs in this drying system, namely the hot air from solar collector and hot air from LPG heating. As shown in both Figures 1 and 2, ambient air passed through the solar collector to be heated, then entered the drying chamber through the air duct at the bottom front of the dryer. At the bottom of the dryer, an air hole with a diameter of 7 cm was connected with the pipes from the blower and burner unit, which carried in hot air from LPG heating. After entering the dryer, both hot air from solar collector and the LPG flew upwards through the drying trays and exited the drying chamber through an air duct at the top.

**Figure 3:**
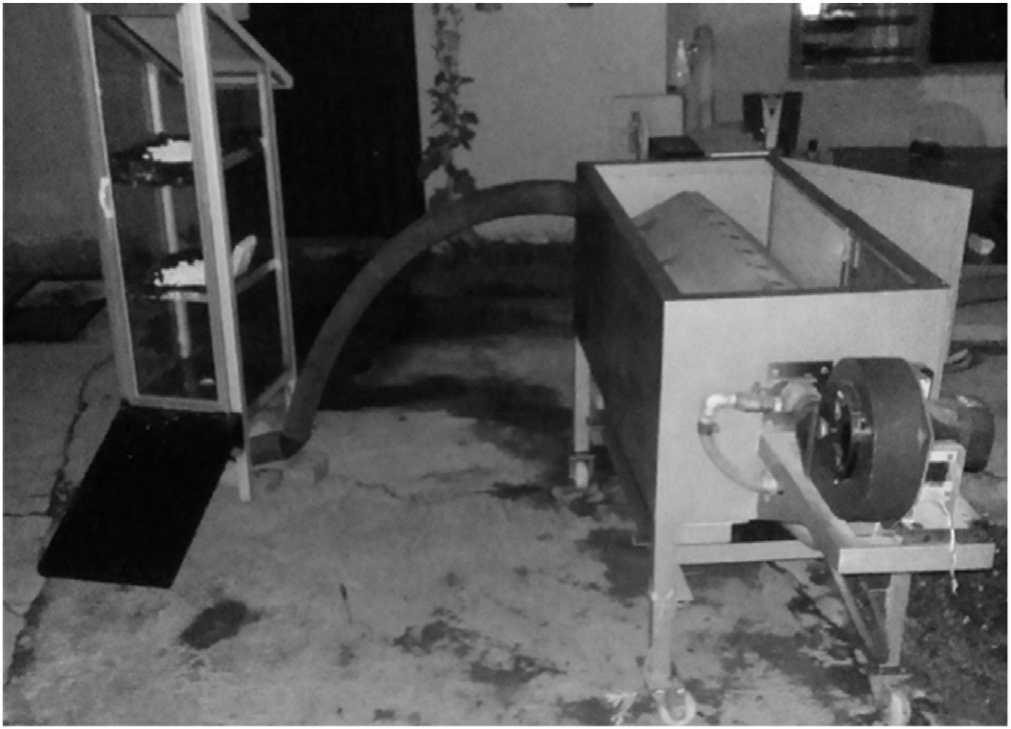
Hybrid solar-dryer system.

**Figure 4:**
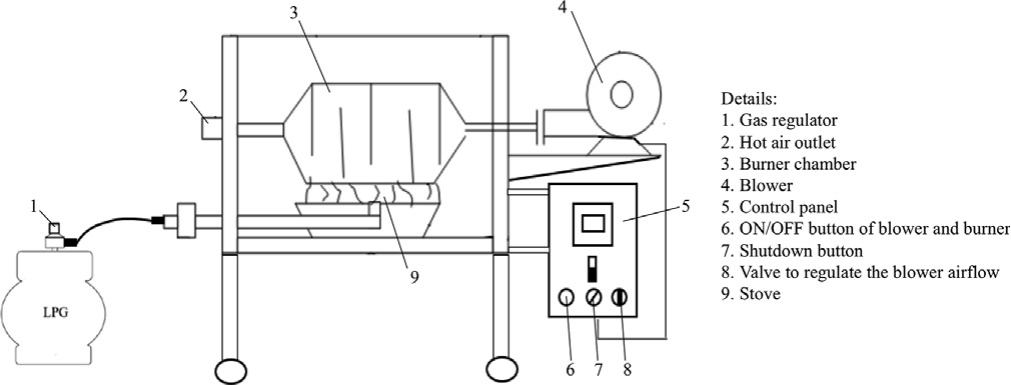
Schematic drawing of blower and burner unit [17].

Figure 3 shows the complete hybrid solar-dryer system, which consists of a drying chamber and blower/burner unit connected to each other. Meanwhile, Figure 4 shows the schematic drawing of blower and burner unit, along with the details of its parts. A 0.37 kW blower was installed to suck in the ambient air, and then blew it to the burner, the latter of which uses 3 kg LPG as the heat source. At the burner unit, a control panel was installed that acted as a temperature indicator. The On/Off switches for the blower and burner were located inside the control panel. Moreover, the gas regulator on the LPG can be adjusted to increase or decrease the gas input to the burner. This adjusting continued until the desired temperature was reached. Herein, heated air flew to the bottom opening of the drying chamber through a connecting pipe with the diameter of 7 cm.

### 2.3 Experimental procedure

Before the drying process was initiated, the initial lime moisture content was determined using the oven method approved by AOAC International [18], obtaining 73% wet basis or 2.7 g H_2_O/g dry lime. The limes were cut horizontally into small slices with a thickness of 2 mm for the ease of placement inside the drying trays. The only variable changed herein was the drying temperature, which ranged from 40 °C to 80 °C with stepwise increments of 10 °C. The drying process was initiated by weighing the wet lime slices: 100 g for each tray, or 200 g for each drying temperature. At the same time, the hybrid solar dryer was prepared by turning on the blower and burner unit, setting up the LPG, and connecting the air pipes from the burner unit to the bottom part of the dryer. Then, the drying air was heated until it reached the desired temperature, using the gas regulator to adjust the amount of gas used to burn the air. After achieving a constant temperature, the lime slices were placed on the top and bottom trays. Lime weight for each tray was measured using digital scales every hour. In addition, the ambient temperature, dryer-inlet temperature, drying-chamber temperature, drying-chamber relative humidity, and solar intensity were also measured every hour using their respective measurement tools. The drying process was conducted for the aforementioned temperatures until there were no significant changes in lime weight for two consecutive hours. Thereafter, the limes were placed in polystyrene bags and stored in the refrigerator.

### 2.4 Data analyses

The recorded data were used to assess the performance of the hybrid solar dryer. The analyses are described in the following subsections.

#### 2.4.1 Moisture content and drying rate analysis

On the basis of the measurements of lime weight, the moisture content of lime at any given time can be determined, as shown in Eq. (1). The moisture content data can be plotted versus the drying time to obtain the moisture content curve. Meanwhile, Eq. (2) shows the formula of drying rate [15].

(1) $$X=\frac{m_{i}-m_{d}}{m_{d}}$$

(2) $$R_{d}=\frac{m_{2}-m_{1}}{t}$$

where *X* denotes the moisture content of lime (g H_2_O/g dry lime), *m*_*i*_ denotes the mass of wet limes at any time during drying (g), and *m*_*d*_ denotes the mass of dry limes (g). *R*_*d*_ denotes the drying rate (g/h) while *m*_*2*_ *– m*_*1*_ denotes the mass difference of lime between two consecutive measurements (g) within the time interval *t* (h).

#### 2.4.2 Dryer efficiency analysis

Hybrid solar-dryer efficiency is defined as the ratio of energy used to evaporate the water and the total energy supplied to the dryer [12, 15].

(3) $$\eta_{d}=\frac{m_{w}.h_{fg}}{IAt+E+m_{fuel}.C_{v}}\times 100$$

where *η*_*d*_ denotes the dryer efficiency (%), *m*_*w*_ denotes the mass of evaporated water (kg), *h*_*fg*_ denotes the latent heat of vaporized water (kJ/kg), *I* denotes the solar intensity (W/m^2^), *A* denotes the solar-collector area (m^2^), *t* denotes the drying time (s), *E* denotes the blower energy consumption (kJ), *m*_*fuel*_ denotes the used-fuel mass (kg), and *C*_*v*_ denotes the heating value of LPG (kJ/kg).

#### 2.4.3 Thin-layer modeling

In this experiment, 10 thin-layer drying models were used to determine the best model to accurately describe the drying behavior of limes using MATLAB software, which were outlined in Table 2. *k* denotes the drying rate constant (s^−1^) while *n*, *a*, *b*, and *c* denotes the dimensionless model constants. Thin-layer modeling was performed for every drying air temperature. Indeed, said models were usually applied for drying fruits and vegetables [19]. Moreover, in said models, a dimensionless new variable was introduced, the moisture ratio (MR), which was defined as [20]:

(4) $$MR=\frac{M-M_{e}}{M_{o}-M_{e}}$$

where *M* denotes the moisture content at any given time, *M*_*o*_ denotes the initial moisture content, and *M*_*e*_ denotes the equilibrium moisture content. However, according to the existing literature, relative humidity varies continuously during drying, and, accordingly, the values of *M*_*e*_ will be relatively small compared with those of *M* and *M*_*o*_ [20, 21]. Therefore, Eq. (4) can be simplified as:

(5) $$MR=\frac{M}{M_{o}}$$

**Table 2:** Thin-layer drying models.

No	Model	Formula
1	Newton [24]	MR = exp (−kt)
2	Page [25]	MR = exp (−kt^n^)
3	Modified Page [26]	MR = exp [−(kt)^n^]
4	Henderson and Pabis [27]	MR = a exp (−kt^n^)
5	Logarithmic [28]	MR = a exp (−kt) + c
6	Midilli *et al.* [29]	MR = a exp (−kt) + bt
7	Demir *et al.* [30]	MR = a exp (−kt)^n^ + b
8	Wang and Singh [31]	MR = 1 + at + bt^2^
9	Modified Midilli *et al.* [32]	MR = a exp (−kt) + b
10	Approximation of Diffusion [33]	MR = a exp (−kt) + (1 − a) exp (−kbt)

The goodness of fit for each model was evaluated using two parameters, namely, the coefficient of determination (*R*^*2*^) and root mean square error (RMSE). Thin-layer drying model with the highest *R*^*2*^ value and lowest RMSE is the most suitable to describe the drying characteristics of lime [22]. *R*^*2*^ and RMSE can be determined using Eqs. (6) and (7) [23]:

(6) $$RMSE=\sqrt{\frac{1}{N}\overset{N}{\underset{i=1}{\sum }}{\left(MR_{exp,i}-MR_{pre,i}\right)}^{2}}$$

(7) $$R2=\frac{{\left[\sum_{i=1}^{N}\left(MR_{exp,i}-\bar{\;MR_{exp}}\right)\left(MR_{pre,i}-\bar{MR_{pre}}\right)\right]}^{2}}{\sum_{i=1}^{N}{\left(MR_{exp,i}-\bar{MR_{pre}}\right)}^{2}\sum_{i=1}^{N}{\left(MR_{pre,i}-\bar{MR_{pre}}\right)}^{2}}$$

where *MR*_*exp*_ denotes the observed MR from the experiment*, MR*_*pre*_ denotes the predicted value of MR using thin-layer models, and *N* denotes the number of observations. The straight line above *MR*_*exp*_ and *MR*_*pre*_ denotes an average value.

Drying characteristics in the falling-rate period can be described using Fick's diffusion equation [34]. Assuming a long drying time, uniform moisture distributions, and slab geometry for limes, Eq. (8) can be applied to determine the value of effective diffusivity [35]:

(8) $$MR=\frac{8}{\pi^{2}\;}exp\left[\frac{D_{eff}\pi^{2}}{4L^{2}}t\right]$$

where *D*_*eff*_ denotes effective diffusivity (m^2^/s), *L* denotes the half-thickness of lime slices (m), *t* denotes the drying time (s), and *π* is a constant. By transforming Eq. (8) into logarithmic form, a new linear equation can be obtained, as shown in Eq. (9). The value of *D*_*eff*_ can be obtained by plotting ln *MR* and *t* on a linear graph. All calculations of *D*_*eff*_ were conducted using Microsoft Excel.

(9) $$\ln\;MR=\ln\frac{8}{\pi^{2}}-\left[\frac{D_{eff}\pi^{2}}{4L^{2}}t\right]$$

The dependence of effective diffusivity on drying temperature can be estimated using simple Arrhenius equation, as shown in Eq. (10) [36].

(10) $$D_{eff}=D_{o}\;\exp\left[-\frac{E_{a}}{RT}\right]$$

where *D*_*o*_ is the constant equivalent to the diffusivity at infinitely high temperature (m^2^/s), *R* is the universal gas constant (8.314 J/mol.K), *E*_*a*_ is the activation energy (kJ/mole), and *T* is the absolute temperature (K). The *E*_*a*_ and *D*_*o*_ were calculated by plotting ln *D*_*eff*_ and 1/*T*, according to Eq. (11), which is obtained by linearizing Eq. (10).

(11) $$\ln\left(D_{eff}\right)=\ln\left(D_{o}\right)-\frac{E_{a}}{RT}$$

#### 2.4.4 Determination of total phenolic content

The total phenolic content was determined using the Folin–Ciocalteau method [37]. Lime samples were mashed up, and aquadest was added to obtain the extract. Next, the extract was dissolved in a water–methanol solution (1:1). Thereafter, 5 mL of Folin–Ciocalteau reagent (diluted with aquadest, 1:10) and 0.5 mL of the extract solution were mixed. The newly formed solution was then incubated for 5 min, and then 4 mL of sodium carbonate 1 M was added; after which point, the solution was further incubated for 15 min. The standard solution used was gallic acid with a concentration of 20–70 μg/mL. The standard solution was also treated similar to the lime samples. The samples and standard solution were measured using colorimetry at 765 nm. The total phenolic content is expressed in terms of total gallic acid equivalent (GAE) per 100 g of dry limes; the values were determined using the gallic acid calibration curve.

#### 2.4.5 Determination of vitamin C content

To determine the total vitamin C content, Tillman's method was used with some modifications [38]. First, the limes were squashed to obtain the extract, which was then filtered to obtain a clear solution, making it easier to take spectroscopy readings. Then, 5 mL of the solution was mixed with 45 mL of aquadest inside a 50 mL flask. The absorbance of the newly formed solution was measured using a spectrophotometer UV/vis with a wavelength of 265 nm. The height of absorbance was measured, and the vitamin C content of the sample was determined using the regression equation from the ascorbic acid standard curve.

## 3 Results and discussion

### 3.1 Analysis of moisture content curve and drying rate curve

**Figure 5:**
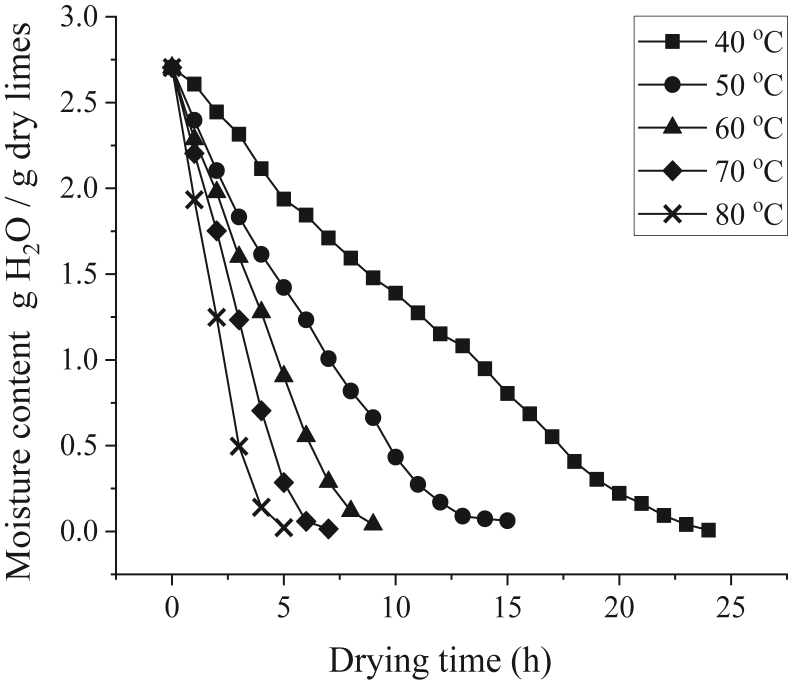
Moisture content curve of lime drying using hybrid solar dryer at the bottom tray.

Figure 5 shows the moisture content curve for limes on the bottom tray at different drying temperatures. The final moisture contents of lime during drying at 40, 50, 60, 70, and 80 °C were 0.0074, 0.063, 0.0407, 0.0148, and 0.0222 g H_2_O/g dry lime, with the drying time of 25 h, 15 h, 9 h, 7 h, and 5 h, respectively. Therefore, 80 °C is the fastest drying temperature, requiring only 5 h, whereas 40 °C requires 24 h. Obviously, using higher temperatures results in faster moisture reduction because more heat is generated to evaporate the water [39]. It is evident from Figure 5 that moisture content decreases over time until it reaches a constant value. Also, moisture content reduction is slower during the end of the drying process. This trend is also found on similar studies such as about lemon drying using hybrid solar dryer [40] and about orange peels drying using forced-convection solar dryer [9]. As the drying process continues, the free moisture content located at the surface of the limes decreases, leaving behind bound moisture content, which is typically much harder to evaporate [14]. Bound moisture content is located at the internal cells of lime, which requires more time to evaporate and therefore, will prolong the drying time [41].

**Figure 6:**
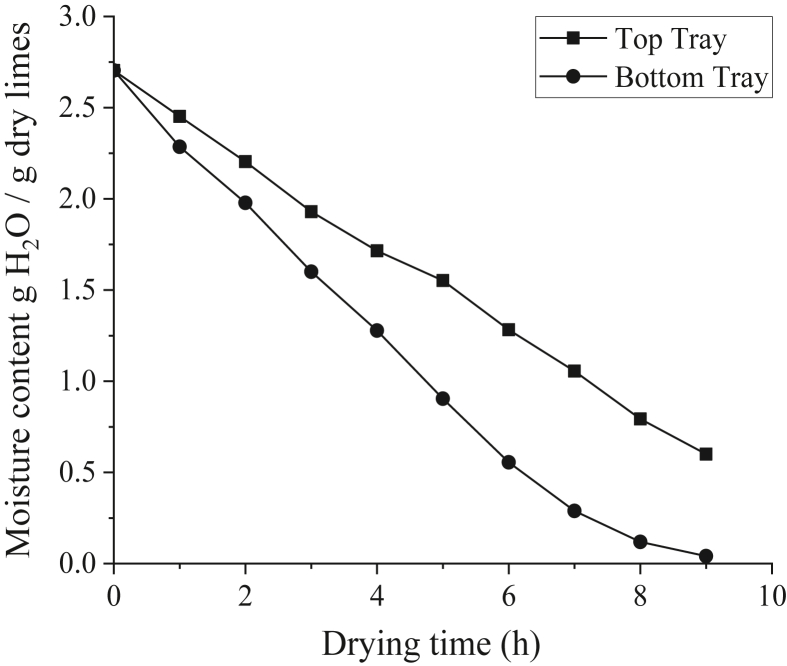
Moisture content curve of lime drying using hybrid solar dryer at different trays.

Figure 6 shows the moisture content curve of limes dried at 60 °C on the top and bottom trays, from which it is evident that the moisture reduction at the bottom tray is faster than that at the top tray. This might be caused by the airflow of the drying air, which flew to the bottom tray first, then flew upwards to the top tray, and finally exited the dryer through the air duct at the top [42]. Therefore, the drying air that flew to the top tray was more humid than that at the bottom tray because it had collected evaporated moisture from limes located at the bottom tray first. As a result, the moisture uptake at the second tray was much slower, thereby slowing the drying process at the top tray [43, 44].

**Figure 7:**
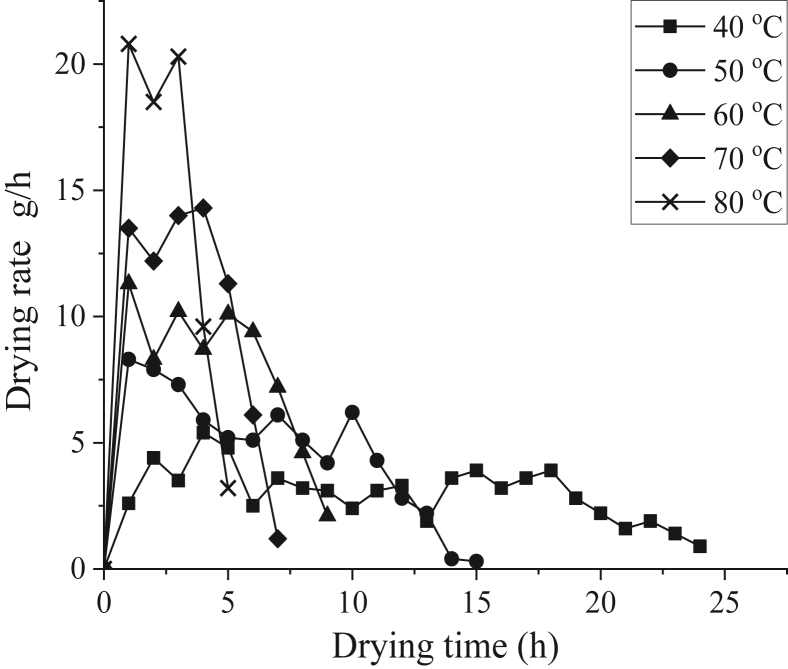
Drying rate curve of limes at the bottom tray at different temperatures.

Figure 7 shows the drying rate curve of limes at the bottom tray at different drying temperatures. The maximum drying rates at 40 °C, 50 °C, 60 °C, 70 °C, and 80 °C were 5.4, 8.3, 11.3, 13.5, and 20.8 g/h, while the average values of drying rate were 3.03, 4.75, 7.99, 10.37, and 14.48 g/h, respectively. It is evident that drying rate will increase if the drying temperature is increased, making the drying process faster, as seen during drying at 80 °C [45, 46]. The drying rates were initially high immediately after the drying process started, and, as drying progressed, the drying rates gradually decreased. If the drying process continued indefinitely, the drying rate will eventually stop decreasing, at which point no weight reduction will occur. This decreasing trend of drying rate over time is also found on other drying research, namely Dissa et al. [47] about Amelie and Brooks mangoes drying using solar dryer, Ayadi et al. [29] about convective drying of spearmint, and Lakshmi et al. [48] about stevia leaves drying using forced-convection solar dryer. Initially, the limes had a high free moisture content, which was easy to evaporate and will eventually depleted as the drying process continued, leaving bound moisture content which was harder to remove, thus decreasing the drying rate [14] This indicated that, aside from drying temperature, drying rate also depends on the total moisture present in the limes and the drying time [49] The decreasing drying rate proves that lime drying occurs during the falling-rate period, which is common for the majority of agricultural products [15, 50].

### 3.2 Analysis of dryer efficiency

**Figure 8:**
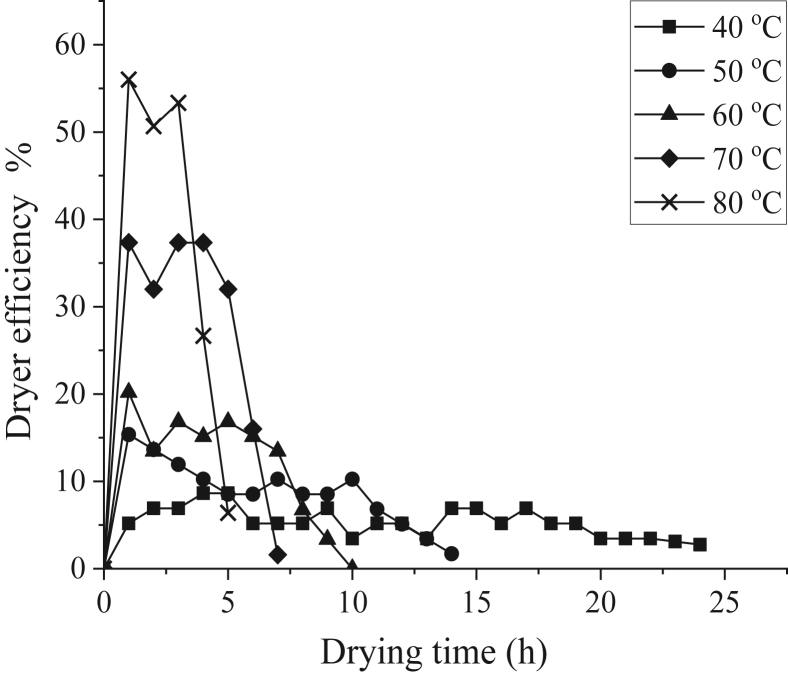
Dryer efficiency at different temperatures.

Figure 8 shows the dryer efficiency during lime drying at different temperatures. The maximum dryer efficiencies at 40 °C, 50 °C, 60 °C, 70 °C, and 80 °C were 6.92%, 15.35%, 20.2%, 37.33%, and 56%, while the average dryer efficiency at said temperatures were 5.36%, 8.77%, 12.11%, 27.66%, and 38.61%, respectively. The energy efficiency values in this experiment are comparable to other solar dryer researches, such as 20%–30% during solar drying of orange peels [9], 30.9%–33.8% during forced-convection solar drying of mango [51], and 22% on average during banana drying using indirect solar dryer [52]. It is evident from Figure 8 that the drying efficiency reached maximum value at the start of the drying process. In other words, dryer efficiency decreases over time, though fluctuations do occur throughout the process. Said fluctuations may be attributed to changes in solar radiation and weather conditions [44, 53]. It can also be seen that increasing drying temperature will increase the dryer efficiency. When the drying temperature increases, the dryer generates more energy, which, in turn, increases the heat and mass transfer of water from the inner part of the limes to the lime surface, resulting in rapid moisture uptake [54]. Conversely, drying at low temperature results in long drying time, making the process inefficient, as seen during drying at 40 °C where the lowest value of dryer efficiency was observed. Therefore, other than increasing the drying temperature, it is possible to increase dryer efficiency by using higher amount of limes, since there will be more water present, which means more heat will be used for evaporation [15, 55]. Meanwhile, Hao et al. [40] suggested that adding insulation measures at the inner drying chamber could increase the dryer efficiency. It can be concluded that, while the value of dryer efficiency found in this experiment are comparable to similar studies, possible suggestions aforementioned above should be considered to improve the dryer performance.

### 3.3 Mathematical modeling

Since examining variables during the drying process can be complicated, mathematical models are used to simulate the distribution of temperature, moisture, and wind velocity. Moreover, said models are helpful with respect to designing and evaluating a drying system, which is important for maintaining food safety and quality [56]. Therefore, understanding the basics of mathematical modeling is important with respect to determine the drying kinetics [19]. Among existing drying models, thin-layer models are able to describe the drying phenomena in a united way, regardless of the controlling mechanism. In addition, thin-layer modeling is relatively simple, and, unlike complex models, the model equations do not require the evaluation of many model parameters [57]. Herein, the moisture content of agricultural products is subjected to a constant relative humidity. Drying conditions, such as temperature, are measured and correlated with the model parameters. The MR data obtained from the experiment were plotted according to 10 thin-layer drying models to determine the most suitable model for lime drying. The thin-layer model with the highest *R*^*2*^ value and lowest RMSE value is optimal. In this experiment, only the drying results at the bottom tray were subjected to mathematical modeling. Table 3 shows the modeling results, including the model parameters as well as the *R*^*2*^ and RMSE values.

**Table 3:** Parameter values from thin-layer modeling.

Models	*T* (°C)	*k*	*n*	*a*	*b*	*c*	*R*^*2*^	RMSE
Newton	40	0.0822					0.9662	0.0780
	50	0.1596					0.9769	0.0608
	60	0.2327					0.9637	0.0793
	70	0.3287					0.9581	0.0890
	80	0.4981					0.9672	0.0757
Page	40	0.0215	1.5454				0.9826	0.0417
	50	0.0568	1.5397				0.9880	0.0392
	60	0.1027	1.5512				0.9893	0.0344
	70	0.1653	1.5679				0.9909	0.0344
	80	0.2726	1.6953				0.9944	0.0278
Modified Page	40	0.0835	1.5965				0.9824	0.0430
	50	0.1556	1.4609				0.9886	0.0355
	60	0.2308	1.4551				0.9884	0.0362
	70	0.3188	1.4389				0.9886	0.0411
	80	0.4704	1.4592				0.9941	0.0292
Henderson and Pabis	40	0.0811	1.0587	1.0902			0.9587	0.0643
	50	0.1239	1.1451	1.0166			0.9830	0.0435
	60	0.2015	1.1044	1.0063			0.9730	0.0649
	70	0.3061	1.1240	1.0506			0.9645	0.0695
	80	0.4184	1.2126	1.0084			0.9850	0.0492
Logarithmic	40	0.0906		1.0646		0.0012	0.9573	0.0703
	50	0.1698		1.0424		0.0011	0.9716	0.0573
	60	0.2462		1.0342		0.0011	0.9581	0.0755
	70	0.3429		1.0250		0.0011	0.9535	0.0862
	80	0.5016		1.0016		0.0011	0.9666	0.0757
Midilli *et al.*	40	0.0902		1.0611	0.0001		0.9574	0.0711
	50	0.1729		1.0567	0.0001		0.9698	0.0574
	60	0.2457		1.0343	0.0001		0.9582	0.0756
	70	0.3323		1.0060	0.0001		0.9569	0.0884
	80	0.5005		1.0022	0.0001		0.9667	0.0757
Demir *et al.*	40	0.0898	0.9978	1.0674	7.97.10^−5^		0.9579	0.0700
	50	0.1635	0.9808	1.0018	9.60.10^−5^		0.9735	0.0636
	60	0.2338	1.0347	0.9921	0.00012		0.9675	0.0753
	70	0.3519	1.0722	1.0110	0.00011		0.9593	0.0786
	80	0.4839	1.0046	1.0162	0.00012		0.9689	0.0730
Wang and Singh	40			−0.0571	0.00065		0.9971	0.0195
	50			−0.1157	0.00333		0.9970	0.0178
	60			−0.1628	0.00587		0.9958	0.0249
	70			−0.2272	0.01196		0.9921	0.0367
	80			−0.3508	0.03058		0.9943	0.0307
Modified Midilli *et al.*	40	0.0806		0.9871	0.00012		0.9678	0.0804
	50	0.1619		1.0097	0.00011		0.9758	0.0596
	60	0.2454		1.0344	0.00011		0.9585	0.0754
	70	0.3289		1.0001	0.00011		0.9580	0.0889
	80	0.5208		1.0275	0.00011		0.9629	0.0740
Approximation of Diffusion	40	0.1127		1.8515	1.53578		0.9766	0.0597
	50	0.2318		1.7942	1.91947		0.9857	0.0381
	60	0.3564		1.9867	1.78562		0.9828	0.0469
	70	0.4710		1.9911	1.60216		0.9791	0.0615
	80	0.7682		2.0874	1.84762		0.9901	0.0386

**Figure 9:**
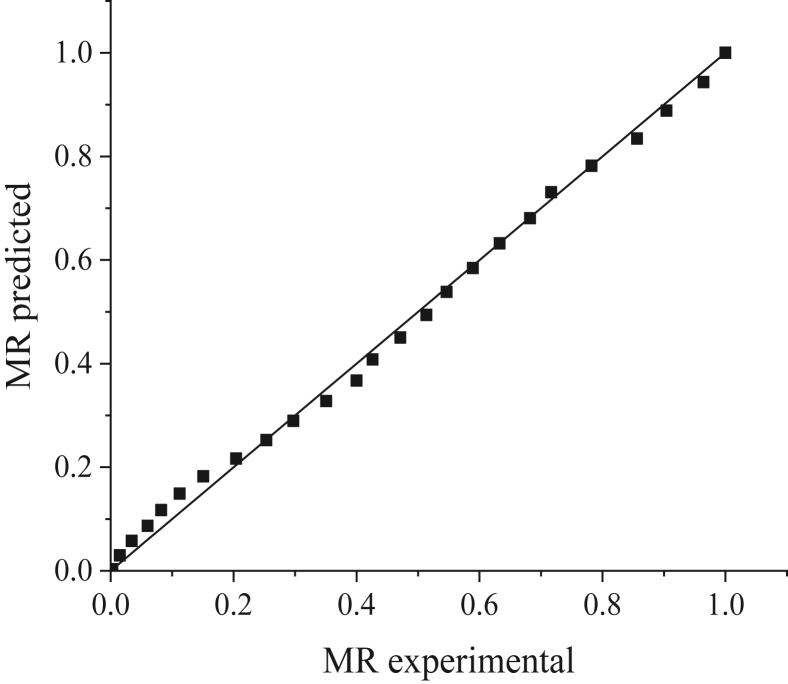
Comparison of MR experimental and MR predicted from the Wang and Singh model at 40 °C.

**Figure 10:**
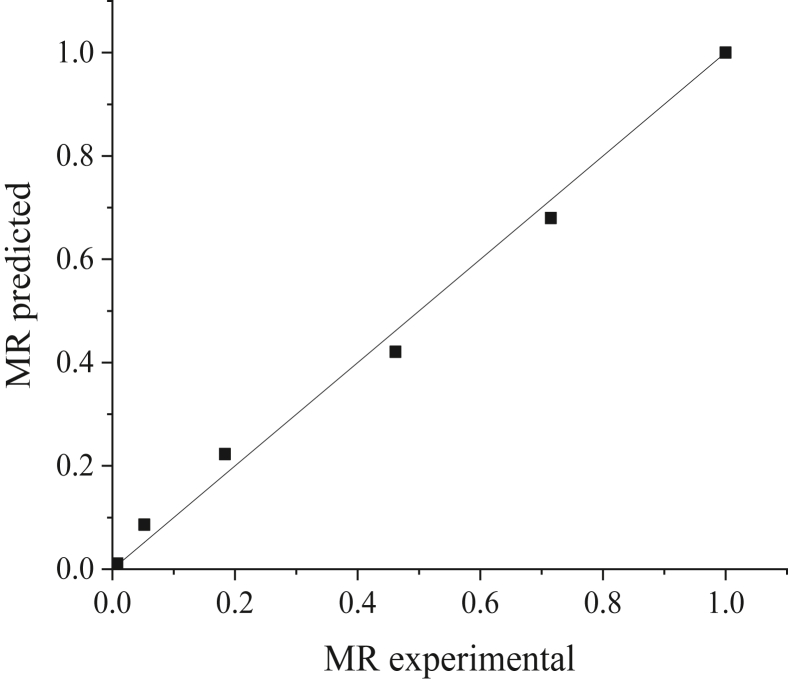
Comparison of MR experimental and MR predicted from the Wang and Singh model at 80 °C.

The results suggest that the values of *R*^*2*^ and RMSE differ randomly and do not follow a specific pattern. After taking the average value of *R*^*2*^ and RMSE for each model at different temperatures, it is evident that the Wang and Singh model has the highest average value of *R*^*2*^ and lowest value of RMSE with 0.9953 and 0.0259, respectively. Therefore, it is the best model to describe the behavior of limes during drying with the hybrid solar dryer. Although the Wang and Singh model has also been used to describe the drying kinetics of fruits such as banana slices [19], it has not been used on limes, and, therefore, we cannot make any comparisons. However, with respect to citrus fruit in general, orange peels have been examined using a natural convection solar tunnel dryer, wherein the Page model was identified as optimal with respect to describing the drying kinetics of orange peels [58]. Another research performed by Hao et al. [40] found that both Two-term model and Wang and Singh model were the most suitable model to describe the drying characteristics of lemon slices under hybrid solar dryer. This differing results can be attributed to several factors, such as drying methods, experimental conditions, and characteristics of the product [19]. Figures 9 and 10 compare the MR values obtained from the experiment with predicted MR values using the Wang and Singh model at 40 °C and 80 °C, respectively. It can be seen from both figures that the values of predicted MR are gathering close to the straight line (experimental data), indicating that the selected model has a good fit with the experimental data.

Assuming a uniform resistance of moisture flow inside the material, the drying of biological products is controlled by liquid/vapor diffusion. Effective diffusivity represents the rate of moisture movement inside a product, ignoring the type of diffusion mechanism [34]. Table 4 shows the effective diffusivity of limes at different drying temperatures, from which it is evident that high temperatures increase the value of effective diffusivity [35]. Indeed, an increase in temperature reduces water viscosity and thus increases the activity of water molecules, which, in turn, increases the diffusion rate. This is because the water molecules are loosely bind to the lime matrix, which, in turn, makes evaporation easier [59]. The values of effective diffusivity in this experiment are within 10^−11^ to 10^−9^ m^2^/s, an acceptable range for agricultural and food products [60].

**Table 4:** Effective diffusivity of limes at different drying temperatures.

Temperature (°C)	Effective diffusivity (m^2^/s)
40	4.90 × 10^−11^
50	5.81 × 10^−11^
60	1.05 × 10^−10^
70	2.15 × 10^−10^
80	2.33 × 10^−10^

**Figure 11:**
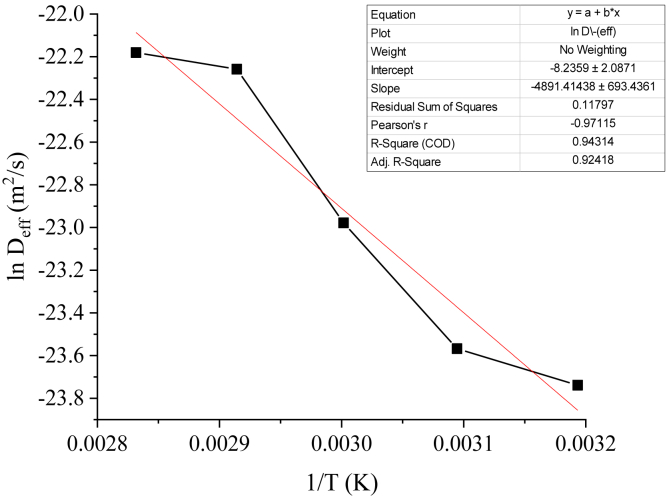
Plot of ln D_eff_ vs 1/T.

Figure 11 shows the plot of ln *D*_*eff*_ and *1/T*. The value of R^2^ of this plot is 0.94314, indicating a fairly good fitting of the prediction. The values of slope (-*E*_*a*_*/R*) and intercept (ln *D*_*o*_) were -4891.414 and -8.2359, respectively. By calculating those values, the diffusivity constant at infinitely high temperature (*D*_*o*_) and activation energy (*E*_*a*_) can be found, with the value of 2.65. 10^−4^ and 40.67 kJ/mol, respectively. It should be noted that activation energy is the minimum energy required to initiate moisture diffusion from the lime [57]. The activation energy value in this experiment are in the range of generally acceptable value of activation energy for most food products, which is 14.42–43.26 kJ/mol [19]. Several studies have calculated the activation energy during the drying process, such as Aviara and Igbeka [60] in their work regarding modelling of cassava starch in a tray dryer (32.993 kJ/mol), Zhang et al. [61] in their work regarding thin-layer modelling of apricot (16.5 kJ/mol), and Shen et al. [36] in their work regarding thin-layer drying of sweet sorghum stalk (21.4 kJ/mol). It can be seen that the activation energy value in this experiment is higher compared to those studies, which may be influenced by several factors such as dryer design, operating temperature, and initial moisture content of the food products [36, 55].

### 3.4 Quality analysis

After the drying process comes to an end, it is necessary to conduct analysis regarding changes in lime quality since drying affects the chemical compounds. The two compounds tested in this experiment were total phenolic content and vitamin C content, the former of which is expressed in terms of total GAE per 100 g of dry limes, with the values determined using the gallic acid calibration curve, which is a plot between concentration and absorbance. As for vitamin C content, the standard curve of ascorbic acid was used as a comparison. Table 5 shows the values of phenolic and vitamin C content at different drying temperatures.

**Table 5:** Phenolic and vitamin C content of limes at different temperatures.

Temperature (°C)	Total phenolic content (mg GAE/100 g dry limes)	Vitamin C (ppm)
40	87.3	0.118
50	70.1	0.077
60	55.2	0.040
70	38.5	0.023
80	27.8	0.015

The initial phenolic content was 116.5 mg GAE/100 g dry limes. From Table 4, it is evident that an increase in drying temperature reduces phenolic content. This is because the drying process destroys polyphenolic compounds. Moreover, since the presence of water assisted in extracting the phenols, the absence of water from the dried lime caused the inner cells of the lime to adhere together, making it difficult to extract the phenols [62]. The initial vitamin C content was 0.22 ppm. The highest vitamin C content is 0.118 ppm at 40 °C, whereas the lowest is 0.015 ppm at 80 °C. In other words, vitamin C content decreases with an increase in drying temperature. This is because vitamin C degrades when exposed to high temperatures or prolonged drying [63]. Therefore, it is preferable to perform low-temperature treatment on crops to prevent the loss of vitamin C [64].

## 4 Conclusions

In this paper, lime drying using hybrid solar drying method have been successfully performed, along with thin-layer modelling to determine lime-drying characteristics and quality analysis to investigate the effect of drying on the quality of lime. There are four key findings from this experiment, namely:

• Hybrid solar dryer was able to dry limes until it was completely dry, with drying at 80 °C, requiring only 5 h, while drying at 40 °C took 24 h to finish. The drying rate analysis suggested that lime drying mostly took place at the falling-rate period.
• Average dryer efficiency increased from 5.36% to 38.61% when the drying temperature was increased from 40 °C to 80 °C. Changes in solar radiation, weather conditions, as well as the amount of samples dried and the insulation on the dryer may affect the dryer efficiency.
• Wang and Singh model had the best fit with respect to the experimental data. The effective diffusivity and activation energy values of lime were within the range of generally acceptable values for agricultural products.
• The phenolic and vitamin C content decreased when the drying temperature was increased from 40 °C to 80 °C.

It can be concluded that hybrid solar dryer is a viable method for lime drying, with acceptable drying time and dryer efficiency, although the degradation of important compounds in lime due to the effect of temperature should be taken into consideration. Further studies regarding the optimization and modification of the hybrid solar dryer are required to preserve the quality of limes while considering the efficiency of the drying process.

## Declarations

### Author contribution statement

Suherman Suherman: Conceived and designed the experiments; Performed the experiments.

Hadiyanto Hadiyanto: Analyzed and interpreted the data; Wrote the paper.

Evan Eduard Susanto: Analyzed and interpreted the data.

Shesar Anis Rahmatullah: Performed the experiments.

Aditya Rofi Pratama: Contributed reagents, materials, analysis tools or data.

### Funding statement

This work was supported by The Faculty of Engineering, 10.13039/501100005844Diponegoro University, Indonesia, through Strategic Research Grant 2019.

### Competing interest statement

The authors declare no conflict of interest.

### Additional information

No additional information is available for this paper.

## References

[1] Aibinu I., Adenipekun T., Adelowotan T., Ogunsanya T., Odugbemi T. (2007). Evaluation of the antimicrobial properties of different parts of citrus aurantifolia (lime fruit) as used locally. Afr. J. Tradit., Complementary Altern. Med..

[2] Ramesh Yadav A., Chauhan A.S., Rekha M.N., Rao L.J.M., Ramteke R.S. (2004). Flavour quality of dehydrated lime [Citrus aurantifolia (Christm.) Swingle]. Food Chem..

[3] Zhao Y., Sun H., Ma L., Liu A. (2017). Polysaccharides from the peels of Citrus aurantifolia induce apoptosis in transplanted H22 cells in mice. Int. J. Biol. Macromol..

[4] Statistics Indonesia (2018). Statistics of Annual Fruit and Vegetable Plants.

[5] Rafiq S., Singh B., Gat Y. (2019). Effect of different drying techniques on chemical composition, color and antioxidant properties of kinnow (Citrus reticulata) peel. J. Food Sci. Technol..

[6] Li L., Zhang M., Chitrakar B., Jiang H. (2020). Effect of combined drying method on phytochemical components, antioxidant capacity and hygroscopicity of Huyou (Citrus changshanensis) fruit. LWT (Lebensm.-Wiss. & Technol.).

[7] Kumar M., Sansaniwal S.K., Khatak P. (2016). Progress in solar dryers for drying various commodities. Renew. Sustain. Energy Rev..

[8] Stojceska V., Atuonwu J., Tassou S.A. (2019). Ohmic and conventional drying of citrus products: energy efficiency, greenhouse gas emissions and nutritional properties. Energy Procedia.

[9] Ben Slama R., Combarnous M. (2011). Study of orange peels dryings kinetics and development of a solar dryer by forced convection. Sol. Energy.

[10] Sun Y., Shen Y., Liu D., Ye X. (2015). Effects of drying methods on phytochemical compounds and antioxidant activity of physiologically dropped un-matured citrus fruits. LWT - Food Sci. Technol..

[11] Imre L., Mujumdar A.S. (2014). Solar drying. Handb. Ind. Dry..

[12] Ndukwu M.C., Simo-tagne M., Abam F.I., Onwuka O.S., Prince S., Bennamoun L. (2020). Exergetic sustainability and economic analysis of hybrid solar-biomass dryer integrated with copper tubing as heat exchanger. Heliyon.

[13] Gudiño-Ayala D., Calderón-Topete Á. (2014). Pineapple drying using a new solar hybrid dryer. Energy Procedia.

[14] Anum R., Ghafoor A., Munir A. (2017). Study of the drying behavior and performance evaluation of gas fired hybrid solar dryer. J. Food Process. Eng..

[15] Murali S., Amulya P.R., Alfiya P.V., Delfiya D.S.A., Samuel M.P. (2020). Design and performance evaluation of solar - LPG hybrid dryer for drying of shrimps. Renew. Energy.

[16] Suherman S., Hadiyanto H., Susanto E.E., Utami I.A.P., Ningrum T. (2020). Hybrid solar dryer for sugar-palm vermicelli drying. J. Food Process. Eng..

[17] Suherman S., Susanto E.E., Zardani A.W., Dewi N.H.R., Hadiyanto H. (2020). Energy–exergy analysis and mathematical modeling of cassava starch drying using a hybrid solar dryer. Cogent Eng.

[18] Nielsen S.S. (2010). Food Analysis.

[19] Onwude D.I., Hashim N., Janius R.B., Nawi N.M., Abdan K. (2016). Modeling the thin-layer drying of fruits and vegetables: a review. Compr. Rev. Food Sci. Food Saf..

[20] Dairo O.U., Aderinlewo A.A., Adeosun O.J., Ola I.A., Salaudeen T. (2015). Solar drying kinetics of cass ava slices in a mixed flow dryer. Acta Technol. Agric..

[21] Sanni L.A., Odukogbe O.O. (2016). Mathematical modelling of thin-layer drying kinetics of cassava meal in a conductive rotary dryer. Adv. J. Food Sci. Technol..

[22] Charmongkolpradit S., Luampon R. (2017). Study of thin layer drying model for cassava pulp. Energy Procedia.

[23] Dhanushkodi S., Wilson V.H., Sudhakar K. (2017). Mathematical modeling of drying behavior of cashew in a solar biomass hybrid dryer. Resour. Technol..

[24] El-Beltagy A., Gamea G.R., Amer Essa A.H. (2007). Solar drying characteristics of strawberry. J. Food Eng..

[25] Tzempelikos D.A., Vouros A.P., Bardakas A.V., Filios A.E., Margaris D.P. (2014). Case studies on the effect of the air drying conditions on the convective drying of quinces. Case Stud. Therm. Eng..

[26] Vega A., Fito P., Andrés A., Lemus R. (2007). Mathematical modeling of hot-air drying kinetics of red bell pepper (var. Lamuyo). J. Food Eng..

[27] Hashim N., Daniel O., Rahaman E. (2014). A preliminary study: kinetic model of drying process of pumpkins (Cucurbita Moschata) in a convective hot air dryer. Agric. Agric. Sci. Procedia..

[28] Kaur K., Singh A.K. (2015). Drying kinetics and quality characteristics of beetroot slices under hot air followed by microwave finish drying. Afr. J. Agric. Res..

[29] Ayadi M., Ben Mabrouk S., Zouari I., Bellagi A. (2014). Kinetic study of the convective drying of spearmint. J. Saudi Soc. Agric. Sci..

[30] Demir V., Gunhan T., Yagcioglu A.K. (2007). Mathematical modelling of convection drying of green table olives. Biosyst. Eng..

[31] Omolola A.O., Jideani A.I.O., Kapila P.F. (2014). Modeling microwave drying kinetics and moisture diffusivity of mabonde banana variety. Int. J. Agric. Biol. Eng..

[32] Gan P.L., Poh P.E. (2014). Investigation on the effect of shapes on the drying kinetics and sensory evaluation study of dried jackfruit. Int. J. Sci. Eng..

[33] Yaldýz O., Ertekýn C. (2001). Thin layer solar drying of some vegetables. Dry. Technol..

[34] Ertekin C., Firat M.Z. (2017). A comprehensive review of thin-layer drying models used in agricultural products. Crit. Rev. Food Sci. Nutr..

[35] Tzempelikos D.A., Vouros A.P., Bardakas A.V., Filios A.E., Margaris D.P. (2015). Experimental study on convective drying of quince slices and evaluation of thin-layer drying models. Eng. Agric. Environ. Food..

[36] Shen F., Peng L., Zhang Y., Wu J., Zhang X., Yang G., Peng H., Qi H., Deng S. (2011). Thin-layer drying kinetics and quality changes of sweet sorghum stalk for ethanol production as affected by drying temperature. Ind. Crop. Prod..

[37] Ghasemi K., Ghasemi Y., Ebrahimzadeh M.A. (2009). Antioxidant activity, phenol and flavonoid contents of 13 citrus species peels and tissues. Pak. J. Pharm. Sci..

[38] Santos D.A., Lima K.P., Março P.H., Valderrama P. (2016). Vitamin C determination by ultraviolet spectroscopy and multiproduct calibration. J. Braz. Chem. Soc..

[39] Suherman S., Hidayati N. (2018). Performance evaluation of pneumatic dryer for aren (arenga piñata) flour. 24th Reg. Symp. Chem. Eng. (RSCE 2017).

[40] Hao W., Liu S., Mi B., Lai Y. (2020). Mathematical modeling and performance analysis of a new hybrid solar dryer of lemon slices for controlling drying temperature. Energies.

[41] Sansaniwal S.K., Kumar M., Rajneesh, Kumar V. (2017). Investigation of indirect solar drying of ginger rhizomes (Zingiber officinale): a comparative study. J. Eng. Sci. Technol..

[42] Suherman S., Djaeni M., Wardhani D.H., Dzaki M.R., Bagas M.N.F. (2018). Performance analysis of solar tray dryer for cassava starch. 24th Reg. Symp. Chem. Eng..

[43] Yassen T.A., Al-Kayiem H.H. (2016). Experimental investigation and evaluation of hybrid solar/thermal dryer combined with supplementary recovery dryer. Sol. Energy.

[44] Baniasadi E., Ranjbar S., Boostanipour O. (2017). Experimental investigation of the performance of a mixed-mode solar dryer with thermal energy storage. Renew. Energy.

[45] Hossain M.A., Amer B.M.A., Gottschalk K. (2008). Hybrid solar dryer for quality dried tomato. Dry. Technol..

[46] Mortezapour H., Ghobadian B., Minaei S., Khoshtaghaza M.H. (2012). Saffron drying with a heat pump-assisted hybrid photovoltaic-thermal solar dryer. Dry. Technol..

[47] Dissa A.O., Bathiebo D.J., Desmorieux H., Coulibaly O., Koulidiati J. (2011). Experimental characterisation and modelling of thin layer direct solar drying of Amelie and Brooks mangoes. Energy.

[48] Lakshmi D.V.N., Muthukumar P., Layek A., Nayak P.K. (2019). Performance analyses of mixed mode forced convection solar dryer for drying of stevia leaves. Sol. Energy.

[49] Sansaniwal S.K., Kumar M. (2015). Analysis of ginger drying inside a natural convection indirect solar dryer: an experimental study. J. Mech. Eng. Sci..

[50] Tsotsas E., Mujumdar A.S. (2011).

[51] Wang W., Li M., Hassanien R.H.E., Wang Y., Yang L. (2018). Thermal performance of indirect forced convection solar dryer and kinetics analysis of mango. Appl. Therm. Eng..

[52] Lingayat A., Chandramohan V.P., Raju V.R.K. (2017). Design, development and performance of indirect type solar dryer for banana drying. Energy Procedia.

[53] Reyes A., Mahn A., Vásquez F. (2014). Mushrooms dehydration in a hybrid-solar dryer, using a phase change material. Energy Convers. Manag..

[54] Aviara N.A., Onuoha L.N., Falola O.E., Igbeka J.C. (2014). Energy and exergy analyses of native cassava starch drying in a tray dryer. Energy.

[55] Balzarini M.F., Reinheimer M.A., Ciappini M.C., Scenna N.J. (2018). Mathematical model, validation and analysis of the drying treatment on quality attributes of chicory root cubes considering variable properties and shrinkage. Food Bioprod. Process..

[56] Castro A.M., Mayorga E.Y., Moreno F.L. (2018). Mathematical modelling of convective drying of fruits: a review. J. Food Eng..

[57] Kadam D.M., Goyal R.K., Gupta M.K. (2011). Mathematical modeling of convective thin layer drying of basil leaves. J. Med. Plants Res..

[58] Pillai B.B.K., Bukke S., Karthikeyan A.K. (2016). Thin layer drying kinetics of mixed mode natural convection solar drying for orange peels. Natl. Conf. Adv. Mech. Eng. (NCAME).

[59] Alara O.R., Abdurahman N.H., Olalere O.A. (2019). Mathematical modelling and morphological properties of thin layer oven drying of Vernonia amygdalina leaves. J. Saudi Soc. Agric. Sci..

[60] Aviara N.A., Igbeka J.C. (2016). Modeling for drying of thin layer of native cassava starch in tray dryer. J. Biosyst. Eng..

[61] Zhang Q.-A., Song Y., Wang X., Zhao W.-Q., Fan X.-H. (2016). Mathematical modeling of debittered apricot ( Prunus armeniaca L .) kernels during thin-layer drying. CyTA - J. Food.

[62] Jahangiri Y., Ghahremanib H., Torghabeh J.A., Salehi E.A. (2011). Effect of temperature and solvent on the total phenolic compounds extraction from leaves of Ficus carica. J. Chem. Pharmaceut. Res..

[63] Ioannou I., Ghoul M. (2013). Prevention of enzymatic browning in fruits and vegetables. Eur. Sci. J..

[64] Mølmann J.A.B., Steindal A.L.H., Bengtsson G.B., Seljåsen R., Lea P., Skaret J., Johansen T.J. (2015). Effects of temperature and photoperiod on sensory quality and contents of glucosinolates, flavonols and vitamin C in broccoli florets. Food Chem..

